# Supplementary material for: De novo protein design of photochemical reaction centers
Source: Nat Commun. 2022 Aug 23;13:4937. doi: 10.1038/s41467-022-32710-5 (PMC9399245; doi:10.1038/s41467-022-32710-5)
Supplement: Supplementary file 1 — Supplementary Information [file 41467_2022_32710_MOESM1_ESM.pdf]

De novo protein design of photochemical reaction centers

Ennist *et al.*

Supplementary Table 1. Amino acid sequences of reaction center maquette and mutants.

RC maquette variant and description	Amino acid sequence (not including N-terminal His_6-tag and TEV protease cleavage sequence)
Reaction Center Maquette<br>(original design)	GSPELRQEHQQLAQEFQQLLQEIQQLGRELLKGELQGIKQLREASEKARNPEKKSVLQKILEDEEKHIELLETLQQTGQ<br>EAQQLLQELQQTGQELWQLGGSGGPELRQKHQQLAQKIQQLLQKHQQLGAKILEDEEKHIELLETILGGSGGDELRELL<br>KGELQGIKQYRELQQLGQKAQQLVQKLQQTGQKLWQLG
L71H mutant<br>(H71-Y168 hydrogen-bonded pair)	GSPELRQEHQQLAQEFQQLLQEIQQLGRELLKGELQGIKQLREASEKARNPEKKSVLQKILEDEEKHIELHETLQQTGQ<br>EAQQLLQELQQTGQELWQLGGSGGPELRQKHQQLAQKIQQLLQKHQQLGAKILEDEEKHIELLETILGGSGGDELRELL<br>KGELQGIKQYRELQQLGQKAQQLVQKLQQTGQKLWQLG
L31D/L71H mutant<br>(increases solvent accessibility to H71-Y168 hydrogen-bonded pair)	GSPELRQEHQQLAQEFQQLLQEIQQLGRELDKGELQGIKQLREASEKARNPEKKSVLQKILEDEEKHIELHETLQQTGQ<br>EAQQLLQELQQTGQELWQLGGSGGPELRQKHQQLAQKIQQLLQKHQQLGAKILEDEEKHIELLETILGGSGGDELRELL<br>KGELQGIKQYRELQQLGQKAQQLVQKLQQTGQKLWQLG
Y168L mutant<br>(Tyr-free control)	GSPELRQEHQQLAQEFQQLLQEIQQLGRELLKGELQGIKQLREASEKARNPEKKSVLQKILEDEEKHIELLETLQQTGQ<br>EAQQLLQELQQTGQELWQLGGSGGPELRQKHQQLAQKIQQLLQKHQQLGAKILEDEEKHIELLETILGGSGGDELRELL<br>KGELQGIKQLRELQQLGQKAQQLVQKLQQTGQKLWQLG
G164C/Y168L mutant<br>(for crosslinking C164-ferrocene to make Fc164)	GSPELRQEHQQLAQEFQQLLQEIQQLGRELLKGELQGIKQLREASEKARNPEKKSVLQKILEDEEKHIELLETLQQTGQ<br>EAQQLLQELQQTGQELWQLGGSGGPELRQKHQQLAQKIQQLLQKHQQLGAKILEDEEKHIELLETILGGSGGDELRELL<br>KGELQCIKQLRELQQLGQKAQQLVQKLQQTGQKLWQLG
Y168C mutant<br>(for crosslinking C168-ferrocene to make Fc168)	GSPELRQEHQQLAQEFQQLLQEIQQLGRELLKGELQGIKQLREASEKARNPEKKSVLQKILEDEEKHIELLETLQQTGQ<br>EAQQLLQELQQTGQELWQLGGSGGPELRQKHQQLAQKIQQLLQKHQQLGAKILEDEEKHIELLETILGGSGGDELRELL<br>KGELQGIKQCRELQQLGQKAQQLVQKLQQTGQKLWQLG
L71H/H124M mutant<br>(ZnP ligated by Met instead of His)	GSPELRQEHQQLAQEFQQLLQEIQQLGRELLKGELQGIKQLREASEKARNPEKKSVLQKILEDEEKHIELHETLQQTGQ<br>EAQQLLQELQQTGQELWQLGGSGGPELRQKHQQLAQKIQQLLQKMQQLGAKILEDEEKHIELLETILGGSGGDELRELL<br>KGELQGIKQYRELQQLGQKAQQLVQKLQQTGQKLWQLG
E34A/L71H mutant<br>(weakens metal binding affinity)	GSPELRQEHQQLAQEFQQLLQEIQQLGRELLKGALQGIKQLREASEKARNPEKKSVLQKILEDEEKHIELHETLQQTGQ<br>EAQQLLQELQQTGQELWQLGGSGGPELRQKHQQLAQKIQQLLQKHQQLGAKILEDEEKHIELLETILGGSGGDELRELL<br>KGELQGIKQYRELQQLGQKAQQLVQKLQQTGQKLWQLG
L31D/E34A/L71H mutant<br>(weakens metal binding affinity; increases solvent accessibility to H71-Y168 hydrogen-bonded pair)	GSPELRQEHQQLAQEFQQLLQEIQQLGRELDKGALQGIKQLREASEKARNPEKKSVLQKILEDEEKHIELHETLQQTGQ<br>EAQQLLQELQQTGQELWQLGGSGGPELRQKHQQLAQKIQQLLQKHQQLGAKILEDEEKHIELLETILGGSGGDELRELL<br>KGELQGIKQYRELQQLGQKAQQLVQKLQQTGQKLWQLG

Supplementary Table 2. Summary of SVD kinetic model analysis of Supplementary Figures 4-11.

Suppl. Figure	31	71	168	Cofactors	Metal	pH	Log k DP*A to DPA	Log k DP*A to DP^+A^-	Log k DP^+A^- to DPA	Log k DP^+A^- to D^+PA^-	Log k D^+PA^- to DPA (Lifetime)	Yield %	Significance
4a	L	L	C	ZnP		7.5	2.5					0	Monad provides P* decay rate
4b	L	L	L	ZnP Heme		7.5	2.5	4.2	not resolved			0	Dyad provides P* to heme ET rate
5a	L	L	C	Fc ZnP Heme		7.5	2.5	4.0	4.9	3.7	0.45 (350 ms)	4.3%	Triad with proton-independent donor
5b	L	L	C	Fc ZnP DADPIX		7.5	2.5	4.1	4.5	4.1	1.7 (20 ms)	31%	Triad with DADPIX acceptor high yield
6a	D	H	Y	Y ZnP Heme		9.5	2.5	4.3	5.4	4.5	2.5 (3.2 ms)	11%	Tyrosinate donor
6b	D	H	Y	Y ZnP Heme		7.5	2.5	4.5	5.9	4.3	4.0 (0.1 ms)	2.4%	Tyrosine donor yield, lifetime drop at pH 7.5
7	L	L	Y	Y ZnP Heme		9.5	2.5	4.3	4.0	2.3	1.4 (40 ms)	2.3%	Without Asp and His Tyrosinate yield drops but lifetime increases
8a	L	H	Y	Y ZnP Heme		9.5	2.5	4.4	4.5	3.0	1.5 (32 ms)	2.9%	His has little effect on Tyrosinate yield or lifetime
8b	L	H	Y	Y ZnP Heme		7.5	2.5	4.2	5.0	3.5	2.7 (2.0 ms)	6%	Asp removal has less effect at neutral pH
9	L	H	Y	Mn Y ZnP Heme	10 μM $MnCl_2$	7.5	2.5	4.1	not resolved	not resolved	2.5 (3.2 ms)	~0	Adding Mn shortens lifetime dramatically
10a	L	H	Y	Fe Y ZnP Heme	10 μM $FeCl_2$	7.5	2.5	4.2	7.5	6.4	0.6 (250 ms)	6.4%	Fe donor increases yield and lengthens lifetime dramatically
10b	L	L	L	Fe ZnP Heme	10 μM $FeCl_2$	7.5	2.5	4.2	not resolved			~0	Removing Tyr disables Fe as donor
11	L	L	Y	Fe Y ZnP CoP	10 μM $FeCl_2$	7.5	2.5	4.0	not resolved			<4%	CoP lower yield acceptor than heme

Visible light-induced difference spectra were captured at multiple time points from 1 μs to ~1 s. Each dataset in this table was collected on a single sample. (Supplementary Table 3 gives mean log rates and quantum yields with standard deviations after repeated experiments). Transient absorption measurements before and after data collection revealed negligible photodegradation over the course of an experiment. Each dataset was analyzed by singular value decomposition (SVD) to fit a simple kinetics model connecting transient spectral species by single exponential rates. SVD analysis extracts the most significant spectral and temporal changes in the 3-D dataset.

Supplementary Figures 4-11 show 8 panels for each analysis: fitted log rates, upper left; log of SVD singular values showing the most important components above the background noise, upper right; light induced difference spectra, upper middle left; the kinetics model fit to the first two SVD component amplitudes for a 3-state model, upper middle right; difference spectra associated with the two or three model kinetic species (the reference ground state being spectrally silent), lower middle left; model state populations as a function of log time, lower middle right. The bottom two panels show a fit of raw absorbance data to P* and iron or cobalt porphyrin redox reference spectra for charge-separation yield estimates.

The kinetics model fit uses a fixed P* to P log rate of 2.5 s^{-1}, as calibrated by the simple exponential decay of the excited Zn porphyrin the absence of a heme electron acceptor and when the heme is pre-reduced before light excitation (Supplementary Fig. 4a). In dyads and triads, charge separation from P*A to P^+A^- takes place over the same edge-to-edge distance as charge recombination from P^+A^- to PA ground state. This charge recombination, with a larger driving force more closely matching the reorganization energy, proceeds rapidly with little accumulation of the P^+A^- intermediate; hence the rate of charge recombination is unresolved. For triads, the difference spectra of intermediate electron-transfer species such as D^+PA^- are expected to be dominated by the heme redox difference spectrum (the spectrum of oxidized heme minus reduced heme) (see Supplementary Figure 1a).

Supplementary Table 3. Statistics for Electron Transfer Rate Measurements in Tyr-ZnP-Heme B Triads in RC Maquette.

Amino acid identity at variable positions					^{3}ZnP*→heme log rate (s^{-1})	pH 9.5 recombination to ground state log rate (s^{-1})	pH 9.5 quantum yield of charge separated state	pH 7.5 recombination to ground state log rate (s^{-1})	pH 7.5 quantum yield of charge separated state
31	34	71	124	168					
L	E	L	H	L	4.2 ± 0.2	—	—	—	—
L	E	L	H	Y	4.1 ± 0.3	0.8 ± 0.1	2.1 ± 0.5%	2.0	1.3%
L	E	H	H	Y	4.2 ± 0.2	1.4 ± 0.2	2 ± 1%	3.0 ± 0.3	7 ± 2%
D	E	H	H	Y	4.5 ± 0.2	2.5 ± 0.1	11.5 ± 0.7%	3.7 ± 0.4	4 ± 2%
L	E	L	M	Y	4.1 ± 0.3	1.0 ± 0.1	8 ± 1%	2.5	11 ± 1%
L	A	H	H	Y	4.6 ± 0.3	1.9	3.4%	—	—
D	A	H	H	Y	4.3	2.0	4.8%	—	—
All*					4.3 ± 0.3, N=40†	1.5 ± 0.6, N=12	5 ± 4%, N=12	3.0 ± 0.7, N=9	6 ± 4%, N=9

* The "All" row gives averaged rates and quantum yields from experiments with different RC maquette mutants. The mutations indicated in this table do not appear to significantly affect the rate of electron transfer from ^{3}ZnP* to heme. However, the large variance in charge recombination rates and quantum yields across different mutants increases the standard deviations for these parameters when values for different mutants are averaged together.

† N value represents the number of separate experiments performed on independently prepared samples to find the indicated mean log rate or quantum yield with its standard deviation.

Supplementary Table 4. Crystallization conditions used to obtain RC maquette crystal structures.

Crystal Structure	RC maquette stock concentration	Cofactors in stock solution*	Well solution	Drop volumes (well solution: protein stock)	Cryoprotectant†
RC maquette, Original design (PDB ID: 5VJS)	12.4 mg/mL (550 μM)	Heme B, ZnP, 1.5 mM $ZnCl_2$	3.6 M NaCl, 100 mM NaAc‡, pH 4.5	1 μL:1 μL	30% glycerol, 3.5 M NaCl, 100 mM NaAc pH 4.4
RC maquette, Original design (PDB ID: 5VJT)	14.2 mg/mL (630 μM)	Heme B, 1.5 mM $ZnCl_2$	2.7 M NaCl, 100 mM NaAc, pH 4.5	1 μL:1 μL	30% glycerol, 3.5 M NaCl, 100 mM NaAc pH 4.4
RC maquette, L71H mutant (PDB ID: 5VJU)	14.2 mg/mL (630 μM)	Heme B	24% w/v PEG§ 1500, 70 mM $CdCl_2$, 100 mM NaAc, pH 4.6	5 μL:1 μL, seeds‖	40% v/v glycerol, 70 mM $CdCl_2$, 100 mM NaAc pH 4.6

* Porphyrin cofactors in stock solution had same molar concentration as protein.
† Crystals were dipped in the indicated cryoprotectant immediately prior to flash freezing in liquid nitrogen or a nitrogen gas stream at 100 K.
‡ NaAc stands for sodium acetate.
§ PEG stands for polyethylene glycol.
‖ Streak seeded with cat whisker from crystals grown with the same protein stock solution hanging over 25% PEG 1500, 80 mM $CdCl_2$, 100 mM NaAc, pH 4.6

Supplementary Table 5. Multiwavelength anomalous dispersion (MAD) statistics for crystal structure 5VJS.

**Measurement**	**Zn K edge**	**Zn K inflection point**	**Remote energy**
Wavelength (Å)	1.2824	1.2831	1.0781
Resolution (Å)	2.1	2.1	2.0
Completeness (%)	99.1	99.1	99.4
I/σ(I)	18.2	19.5	16.8
R_{merge} (%)	6.6	6.3	8.5

Supplementary Table 6. X-ray crystallographic data collection and refinement statistics.

Protein	RC maquette (original design)	RC maquette (original design)	RC maquette (L71H mutant)
PDB ID	5VJS	5VJT	5VJU
Cofactors	Heme B<br>ZnP<br>Tyr<br>Zn(II)	Heme B<br>--<br>Tyr<br>Zn(II)	Heme B<br>--<br>H-bonded His-Tyr<br>Cd(II)
**Data Collection**			
Space group	$P4_12_12$	$P4_12_12$	$P2_1$
X-ray source	NSLS X6A Beamline	NSLS X6A Beamline	Rotating Anode Cu K_α
Wavelength (Å)	1.0781	1.0781	1.54178
Unit cell dimensions			
a (Å)	43.749	43.747	44.48
b (Å)	43.749	43.747	25.88
c (Å)	245.74	245.52	73.71
α (°)	90	90	90
β (°)	90	90	103.64
γ (°)	90	90	90
Resolution (Å)	61.43 - 2.00 (2.05 – 2.00)*	61.38 - 1.45 (1.49 – 1.45)	71.54 - 2.02 (2.07 – 2.02)
R_{merge} (%)	8.5 (97.0)	9.9 (133.7)	5.1 (10.1)
I/σ(I)	16.80 (2.52)	15.30 (1.26)	25.98 (9.46)
Completeness (%)	99.4 (98.8)	99.7 (96.1)	97.3 (71.0)
Redundancy	7.6 (7.4)	8.8 (5.2)	6.7 (3.3)
**Refinement**			
Resolution	61.43 – 2.00 (2.05 – 2.00)	41.21 – 1.45 (1.49 – 1.45)	71.63 – 2.08 (2.13 – 2.08)
Unique reflections	16,280 (1,131)	41,378 (2,848)	9,587 (643)
R_{work}/R_{free}	0.235/0.278	0.202/0.236	0.183/0.243
No. of non-H atoms			
Protein	1585	1585	1587
Ligand	88	48	50
Water	39	172	88
Mean B factor ($Å^2$)	40.77	23.27	31.83
R.m.s. deviations			
Bonds (Å)	0.018	0.031	0.015
Angles (°)	1.829	2.236	1.782

* Values in parentheses represent statistics from the highest resolution shell.

Each dataset was collected on a single crystal.

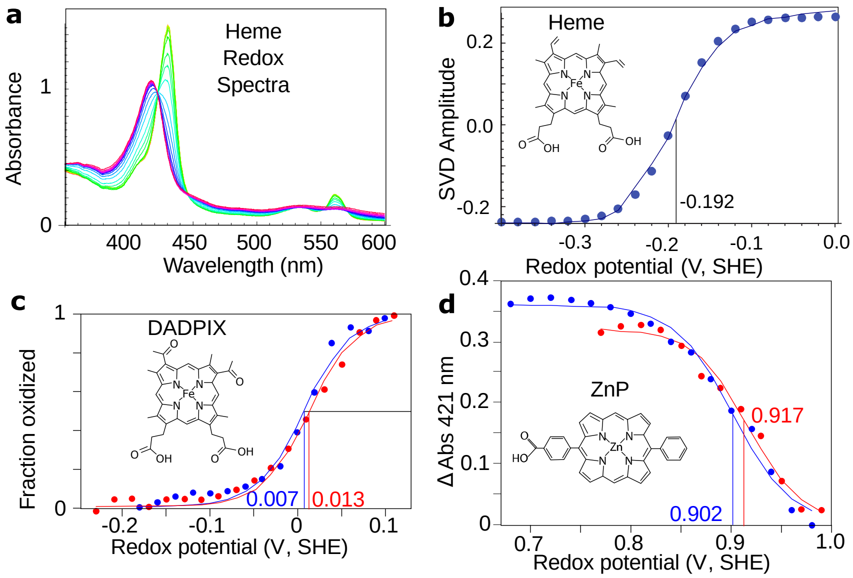

Supplementary Figure 1. Redox midpoint potentials of cofactors. a. Spectra for electrochemical determination of E_m values of heme B in reaction center maquette. Buffer conditions: 15 mM NaCl, 10 mM phosphate buffer, pH 7. **b.** SVD fit of the spectra in panel (a) to a single n=1 Nernst redox transition give a midpoint potential of -0.192 V vs. SHE. **c.** Spectro-electrochemical determination of E_m value of Fe porphyrin DADPIX in reaction center maquette. Buffer conditions: 50 mM phosphate, 200 mM NaCl, pH 7. **d.** Spectro-electrochemical determination of E_m value of ZnP in reaction center maquette. Buffer conditions: 50 mM phosphate, 200 mM NaCl, pH 7.

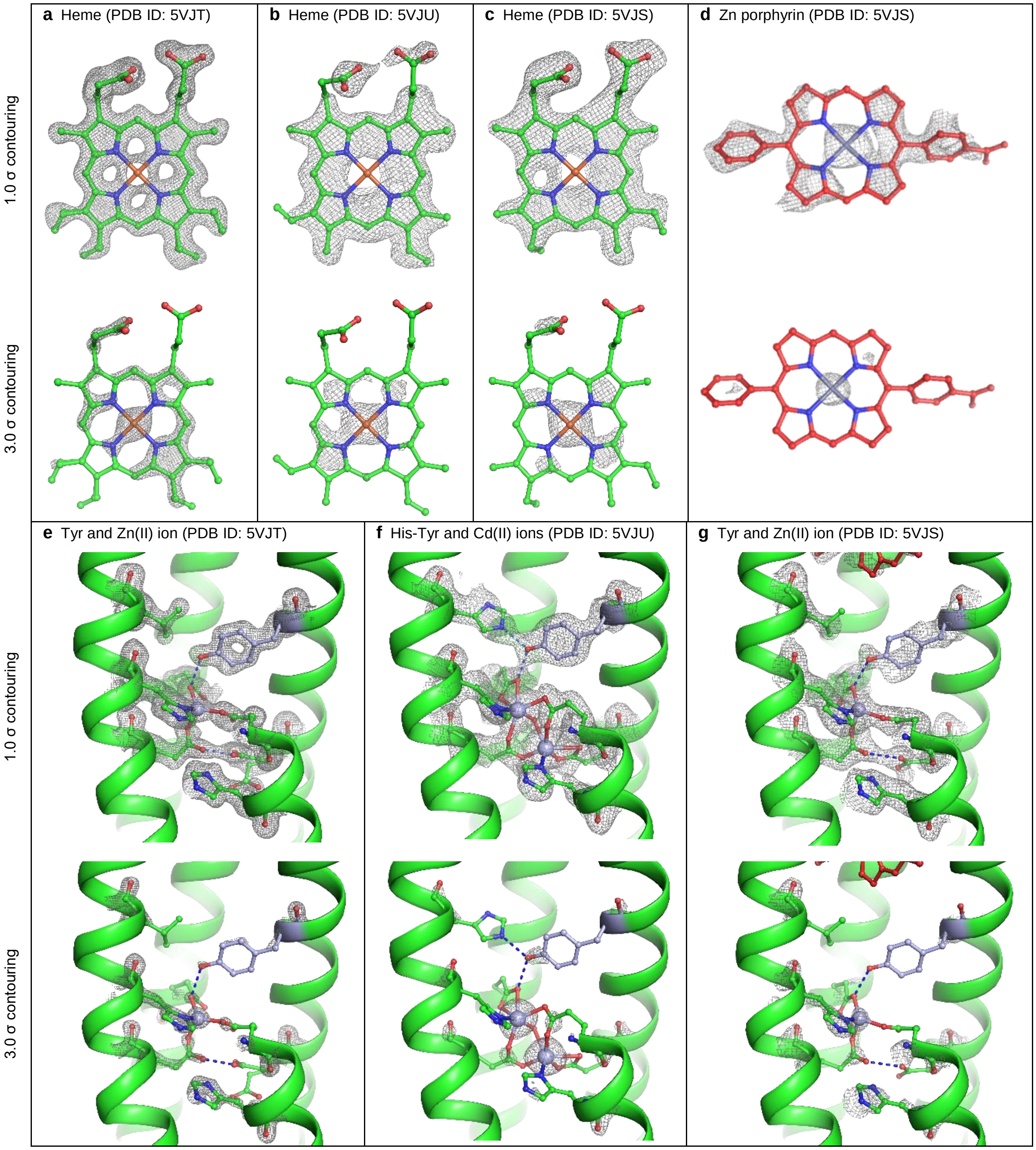

Supplementary Figure 2. 2Fo-Fc omit maps of cofactors. Omit maps of cofactors at 1.0 σ and 3.0 σ contouring levels (grey mesh) were created in PHENIX using simulated annealing to remove model bias. **a-c.** Omit maps of heme B electron acceptor in each of the three crystal structures (PDB IDs: 5VJT, 5VJU, and 5VJS). Heme B has excellent omit map electron density particularly in structure 5VJT, in which it is modeled in two superposed conformations, giving 4 vinyl groups in the model. **d.** The ZnP pigment omit map in structure 5VJS. The ZnP omit map has slightly lower quality that the heme cofactor in the same crystal structure, and the ZnP is modeled with only 70% occupancy. The position of the central Zn ion in the ZnP ligand was confirmed by MAD. **e-g.** Omit maps of electron donor sites. Structures 5VJT and 5VJS (panels (e) and (g)), contain only the Zn(II) ion in the metal site nearest the pigment site, but 5VJU (panel (f)) has two Cd(II) ions bound, as designed. Blue dashes indicate hydrogen bonds.

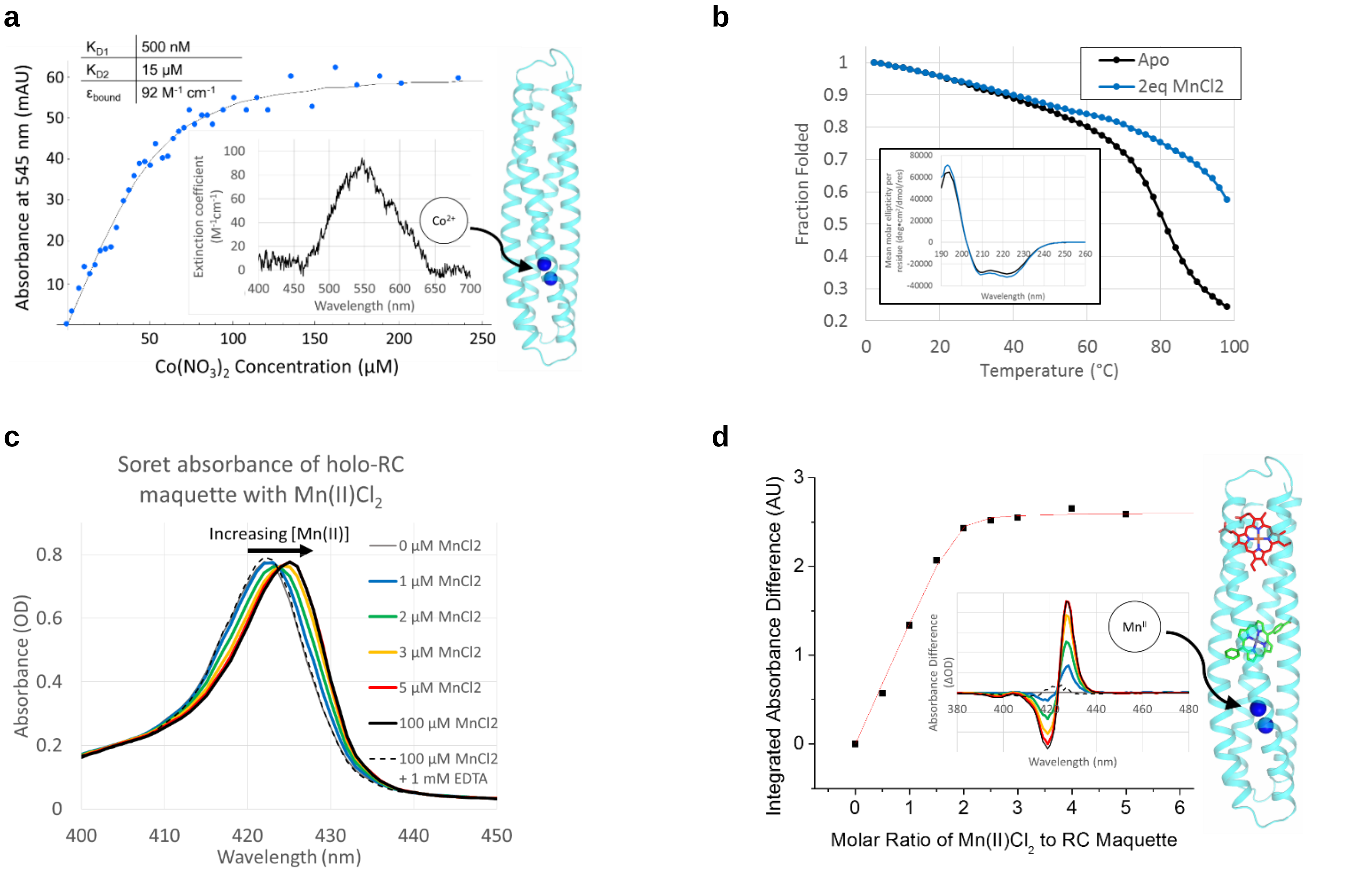

Supplementary Figure 3. Metal binding in RC maquette. a. Spectroscopic titration shows pentacoordinate Co(II) binding, as intended. Stock solution of 17 mM Co(II)$(NO_3)_2$ in water was titrated into 34 µM RC maquette (without porphyrin cofactors) in 10 cm cuvette. The absorbance maximum at 545 nm with an extinction coefficient of 92 $M^{-1}cm^{-1}$ is consistent with 5-coordinate Co(II) binding[1-3]. Fitting titration curve to two-site binding equation[4] yielded stoichiometry of two Co(II) ions per RC maquette and K_Ds of 500 nM and 15 µM for the first and second Co(II) ions, respectively. These values are comparable to Co(II) binding in Due Ferri proteins and bacterioferritin[2,3]. **b.** Thermal denaturation curves estimated from circular dichroism (CD) ellipticity at 222 nm demonstrate RC maquette stabilization upon addition of $MnCl_2$. Black data points represent apo-RC maquette (15.2 µM); blue data points represent RC maquette with two equivalents of $MnCl_2$ per protein (15.2 µM protein and 33 µM $MnCl_2$). CD measurements were performed in 15 mM NaCl, 10 mM phosphate buffer at pH 7. RC maquette melting temperature increases from 82°C in apo-state to >100°C in Mn-bound state. Inset: CD spectra at 25°C prior to thermal denaturation indicate slight increase in helicity upon Mn(II) binding. **c**. Ultraviolet/visible spectra show that porphyrin Soret band shifts from 422 nm to 425 nm upon Mn(II) binding (2 µM RC maquette-ZnP-heme B complex, 50 mM NaCl, 10mM MOPS buffer at pH 7.5). **d.** Band shift in panel (c) was quantified with respect to Mn(II) concentration using difference spectra. The 0 µM $MnCl_2$ spectrum in part (c) was subtracted from each spectrum containing $MnCl_2$, and the sum of the absolute values of absorbance difference from 405 to 440 nm are plotted against the molar ratio of Mn(II) to RC maquette-ZnP-heme B complex. Inset: the aforementioned difference spectra. (Trace colors have same meaning as in panel (c)). Porphyrin Soret bathochromic shift reaches maximum when 2 Mn(II) ions per protein are present, indicating that 2 Mn(II) ions bind per protein, as intended. Addition of excess EDTA chelating agent reverses the spectroscopic transition, confirming that Mn(II) is responsible for the band shift.

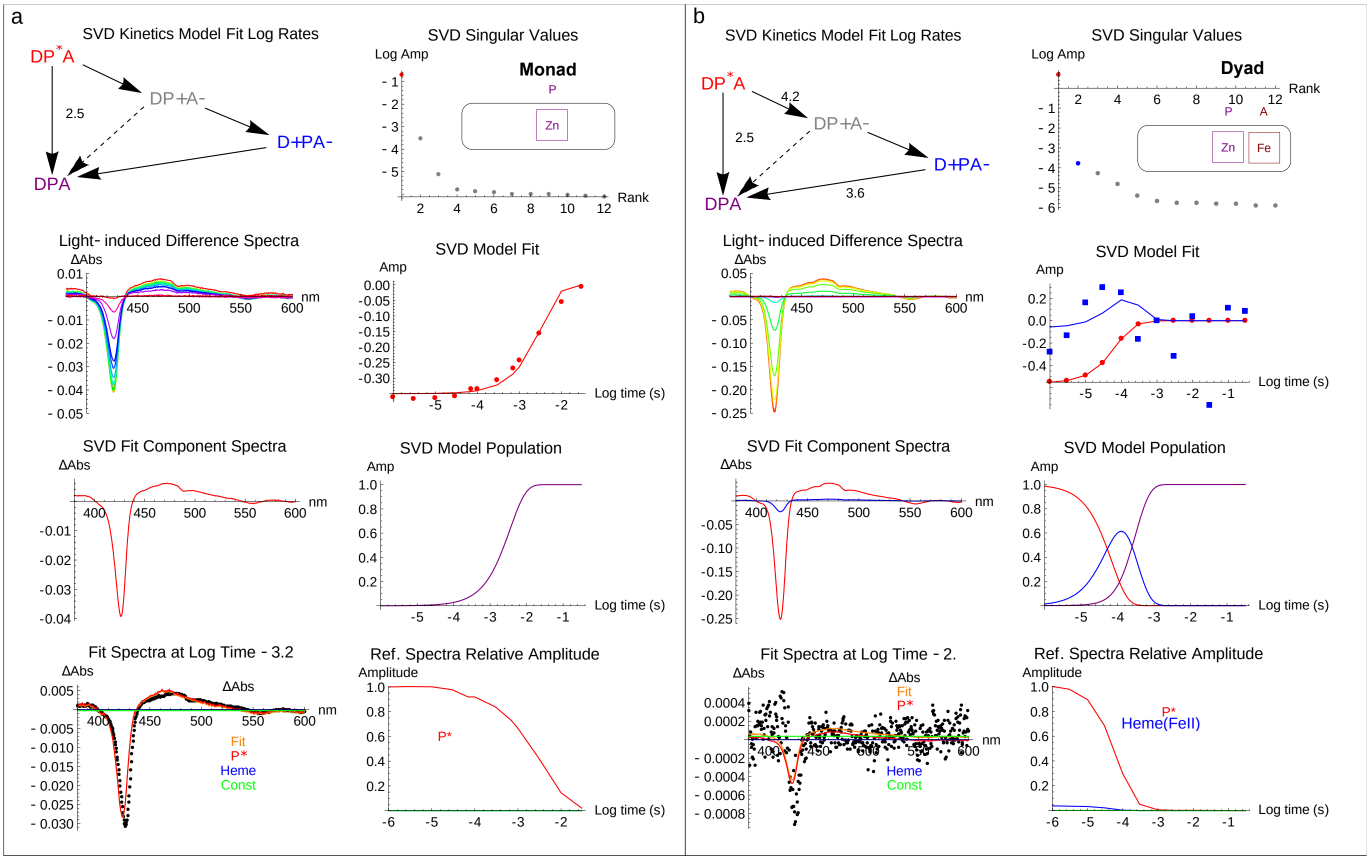

Supplementary Figure 4. Transient absorption kinetics of Monad and Dyad. a. ZnP monad construct at pH 7.5 allows characterization of the spectrum and dynamics of the ZnP* excited triplet state. SVD is dominated by a single component (upper right, red). The time variation of this component (upper middle right) is well described by a single exponential decay with a log rate of 2.5 s^{-1} (3 ms lifetime). The SVD kinetics model fit (upper middle right) with time dependent state populations (lower middle right) generates a spectrum of this component (lower middle left) with Soret band ground state bleach and the broad absorbance from 450 to 600 nm expected for the triplet state. A non-SVD fit of the raw absorbance data to a P* spectrum is shown at lower left and right. **b.** A dyad construct with ZnP and heme B is assembled in the Y168L mutant at pH 7.5. A 2-state kinetic model of the SVD data in which k A^- to P^+ is much faster than k P* to A gives log k P* to A of 4.2 s^{-1}. Sub-panels are as described in the legend of Supplementary Table 2. For consistency in data analysis, here we force a 3-state model upon the dyad system even though no electron donor, D, is present. Consistent with the expectation that heme reduction is transient in the dyad due to fast recombination in the absence of D, a heme redox signal is not observed directly. The dataset is dominated by the P* signal, and log k A^- to P^+ cannot be estimated independently. Even small deviations from single exponential log k P* to A behavior compromise 3-state kinetic model fits. The short lifetime of the charge-separated state prevents an accurate charge separation yield estimate. However, the fact that the P* lifetime in the dyad is 50-fold shorter than in the monad without any evidence of a 3 ms lifetime component of P* decay suggests that nearly all P* reduces A.

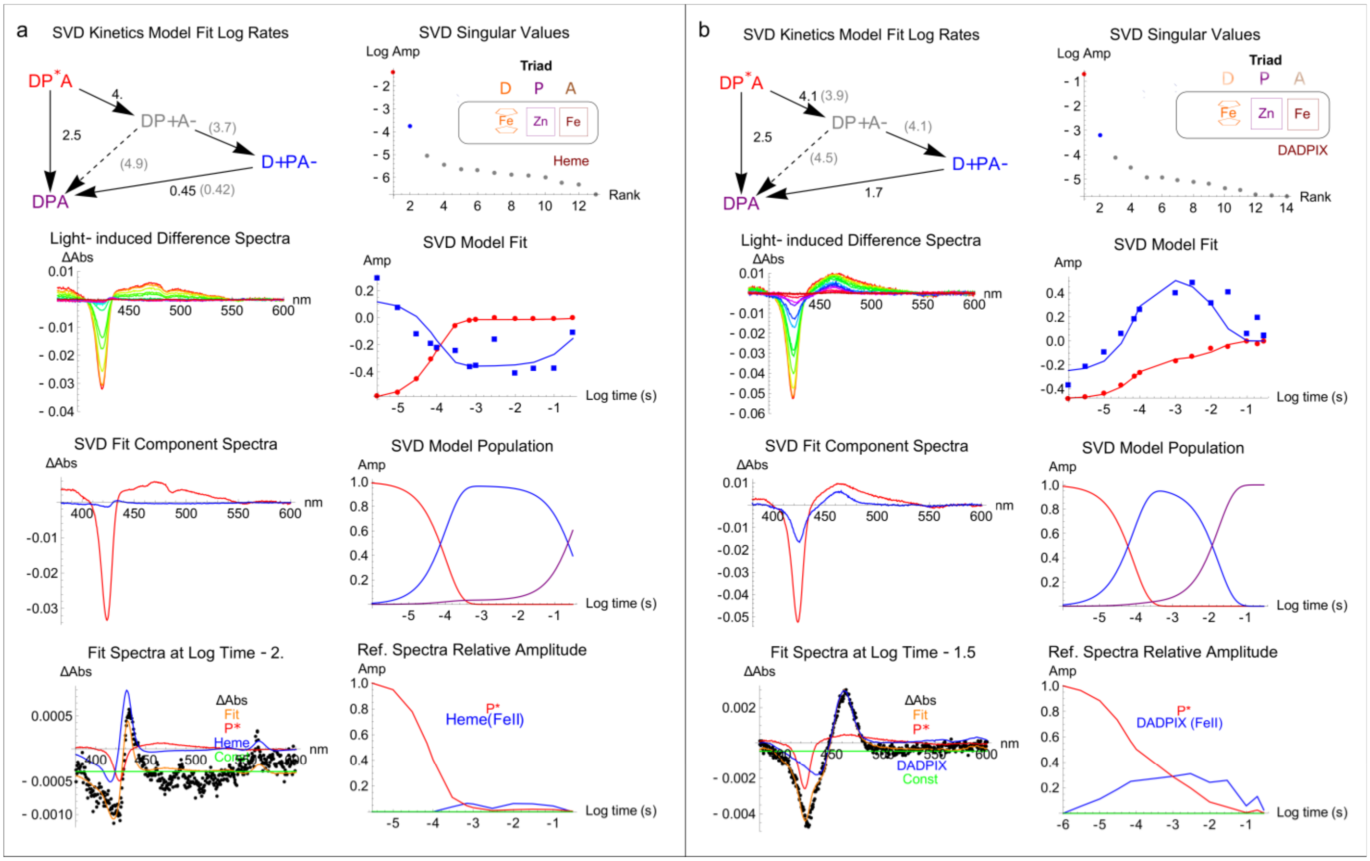

Supplementary Figure 5. Transient absorption kinetics of Ferrocene Triads. a. Ferrocene-ZnP-Heme Triad in Y168C mutant at pH 7.5. SVD indicates a second kinetic species above the background noise (upper right). A 3-state kinetic model fit to the SVD in which k A$^-$ to P$^+$ is much faster than k P* to A gives log k P* to A of 4.0 s^{-1} and a D$^+$PA$^-$ spectrum with a clear heme redox signal (blue), providing direct spectroscopic evidence of heme reduction. Decay of this heme redox signal back to the ground state DPA has a log rate of 0.45 s^{-1}. The yield of heme reduction of ~4% is estimated by fitting P* and heme redox difference spectra to each time trace (lower left and right). A 4-state SVD model fit using this yield gives the log rates in parentheses (upper right). **b.** Ferrocene-ZnP-DADPIX Triad in Y168C mutant at pH 7.5. SVD indicates a second kinetic species above the background noise (upper right). A 3-state kinetic model in which k A$^-$ to P$^+$ is much faster than k P* to A gives log k P* to A of 4.1 s^{-1} and a D$^+$PA$^-$ spectrum with a clear DADPIX redox signal (blue), providing direct evidence of DADPIX reduction. Decay of this state back to the ground state DPA has a log rate of 1.7 s^{-1}. The larger yield of DADPIX reduction of ~31% is estimated by fitting P* and heme redox difference spectra to each time trace (lower left and right). A 4-state SVD model fit using this yield gives the log rates in parentheses (upper left).

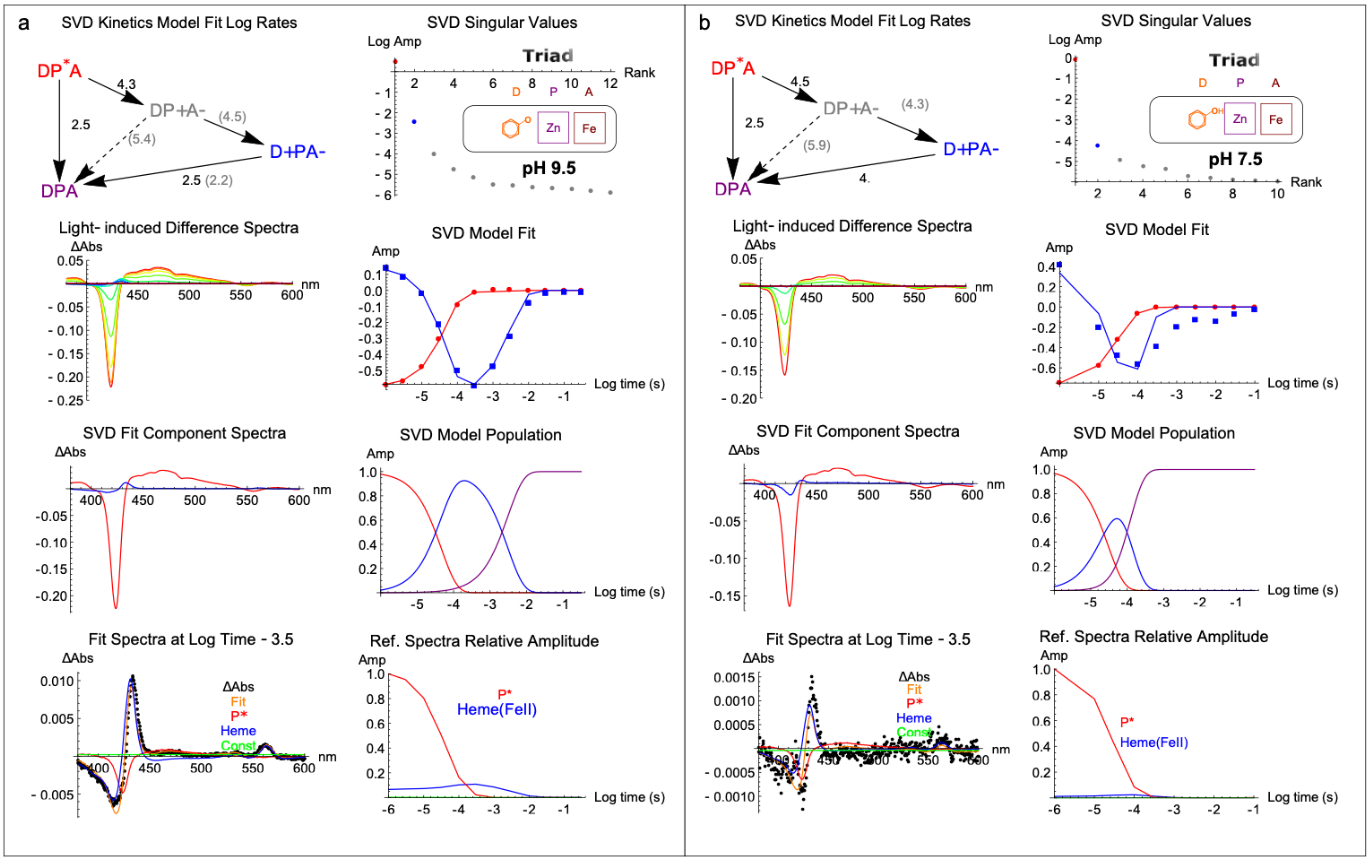

Supplementary Figure 6. Transient absorption kinetics of Tyrosine-ZnP-Heme Triad in L31D/L71H mutant. a. pH 9.5. SVD indicates a second kinetic species above the background noise. A 3-state kinetic model in which k A^- to P^+ >> k P to A gives log k P to A of 4.3 s^{-1} and a D^+PA^- spectrum with a clear heme redox signal (blue), providing direct evidence of heme reduction. Introducing an aspartic acid at L31D dramatically improves the quantum yield of charge separation to 11% compared to Supplementary Fig. 8a. Using this yield in a 4-state SVD model gives log rates shown in parentheses. Decay of this state back to the ground state DPA has a log rate of 2.5 s^{-1} (a 3 ms lifetime of charge separation). **b**. At pH 7.5. SVD indicates a small second kinetic species above the background noise. A 3-state kinetic model in which k A^- to P^+ >> k P to A gives log k P to A of 4.5 s^{-1} and a D^+PA^- spectrum with a heme redox signal (blue) with some contamination from the ZnP triplet bleach. Decay of this state back to the ground state DPA has a log rate of 4.0 s^{-1}. Fit to reference P* and heme redox spectra estimates yield at 2.4%. Using this yield in a 4-state SVD model gives log rates shown in parentheses. Dropping the pH from 9.5 to 7.5 lowers the charge separation yield and shortens the lifetime.

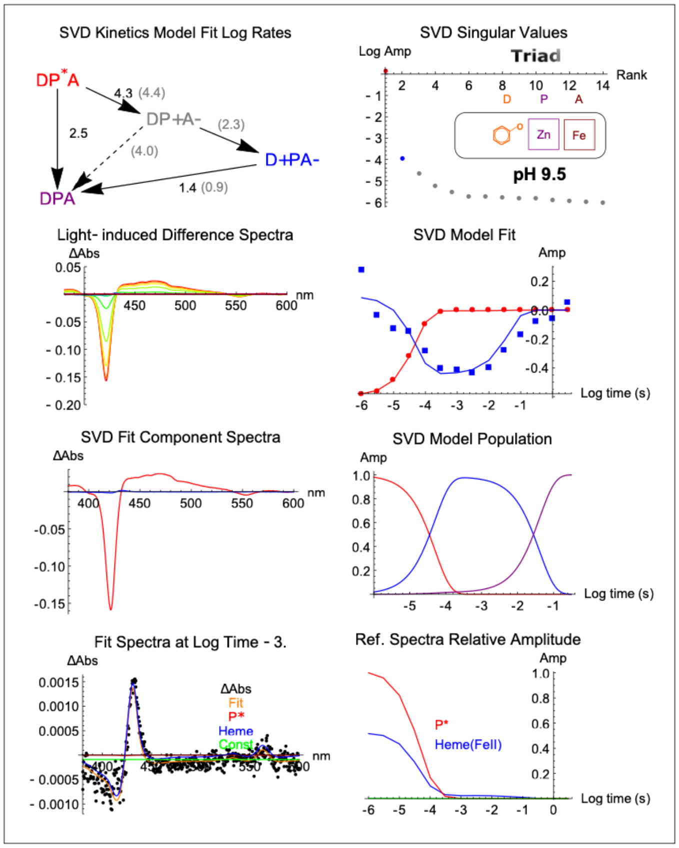

Supplementary Figure 7. Transient absorption kinetics of Tyrosine-ZnP-Heme Triad in RC maquette (original design). a. At pH 9.5, SVD indicates a second kinetic species above the background noise. A 3-state kinetic model in which k A^- to P^+ >> k P to A gives log k P to A of 4.3 s^{-1} and a D^+PA^- spectrum with a clear heme redox signal (blue), providing direct spectral evidence of heme reduction. Fit of the raw absorbance data with P* and heme redox reference spectra estimates yield at 2.3%. Using this yield in a 4-state SVD model gives log rates shown in parentheses. Without Asp31 and His71, the yield of tyrosinate oxidation drops but lifetime increases compared to Supplementary Fig. 6a. Decay of the charge separated state back to ground DPA has a log rate of 1.4 s^{-1}.

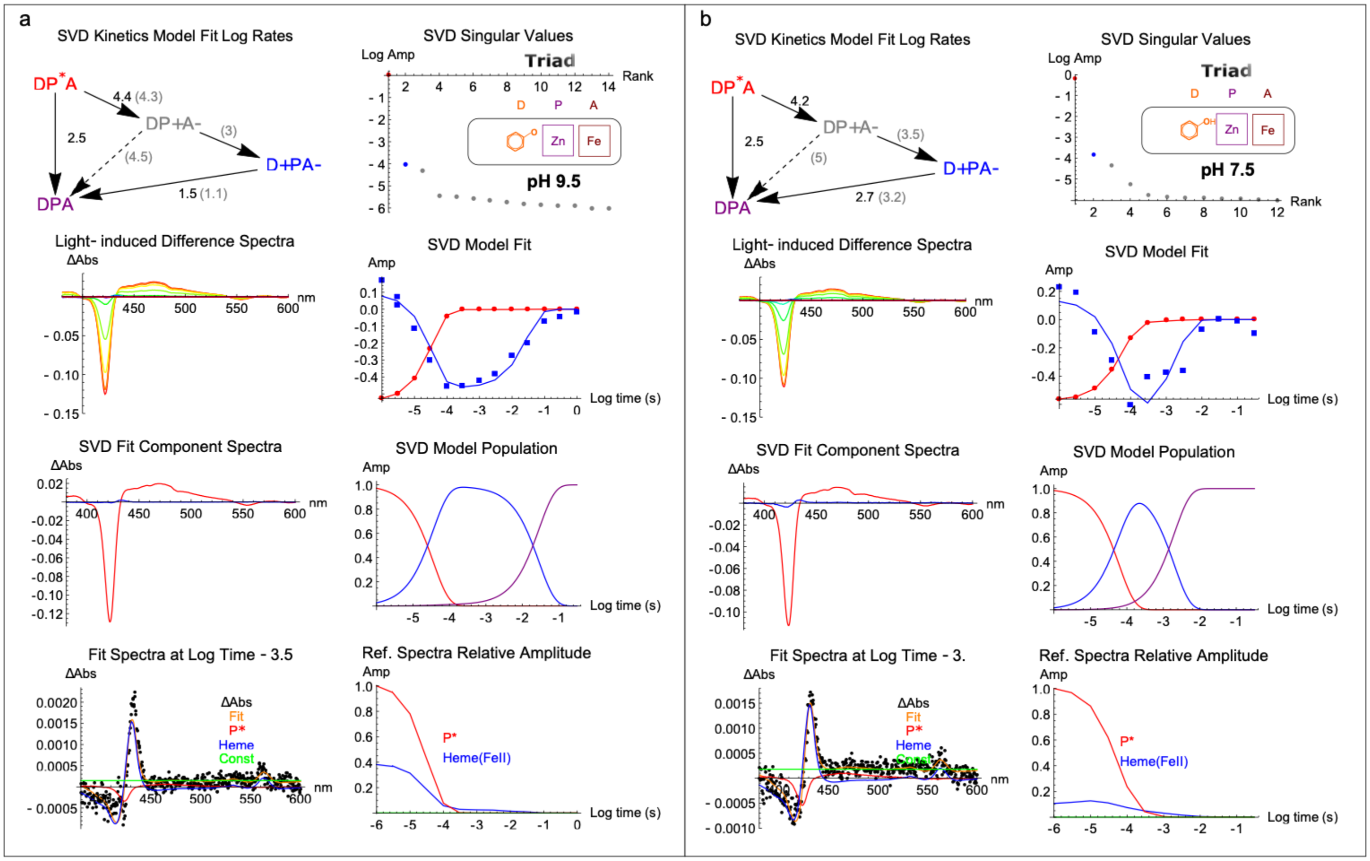

Supplementary Figure 8. Transient absorption kinetics of Tyrosine-ZnP-Heme Triad in L71H variant. a. At pH 9.5, SVD indicates a second kinetic species above the background noise. A 3-state kinetic model in which k A^- to P^+ is much faster than k P to A gives log k P to A of 4.4 s^{-1} and a D^+PA^- spectrum with a clear heme redox signal (blue). Fits to P* and heme redox reference spectra estimate the charge-separation yield at ~2.9%. This yield is used to calculate the rates given in parentheses of a 4-state kinetic model. The log rate of charge recombination from the D^+PA^- state to DPA ground state is 1.5 s^{-1}. **b.** At pH 7.5, a 3-state kinetic model in which k A^- to P^+ is much faster than k P to A gives log k P to A of 4.2 s^{-1} and a D^+PA^- spectrum with a clear heme redox signal (blue). Decay of this state back to the ground state DPA has a log rate of 2.7 s^{-1}. The yield of heme reduction relative to the initial P* triplet state is ~6%.

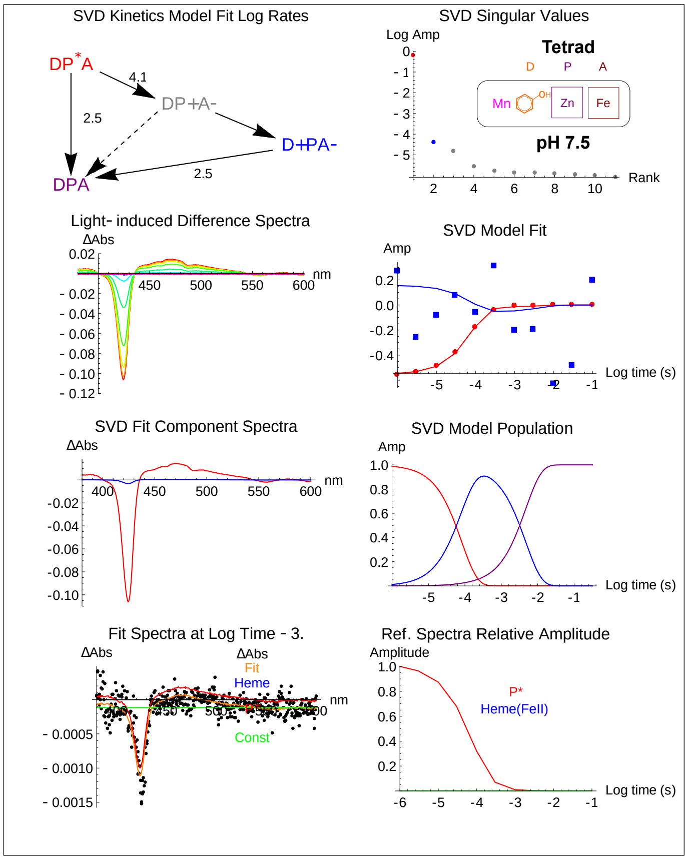

Supplementary Figure 9. Transient absorption kinetics of Mn-Tyr-ZnP-Heme Tetrad in L71H mutant at pH 7.5. With 10 μM $MnCl_2$ present, SVD indicates a second kinetic species barely above the background noise. A 3-state kinetic model in which k A^- to P^+ is much faster than k P to A gives log k P to A of 4.1 s^{-1} and a D^+PA^- spectrum without a clear heme redox signal. A charge separation quantum yield estimate is not possible. With low yield, an estimate of the decay of the charge separated state back to the ground state DPA of 2.5 s^{-1} is not well determined.

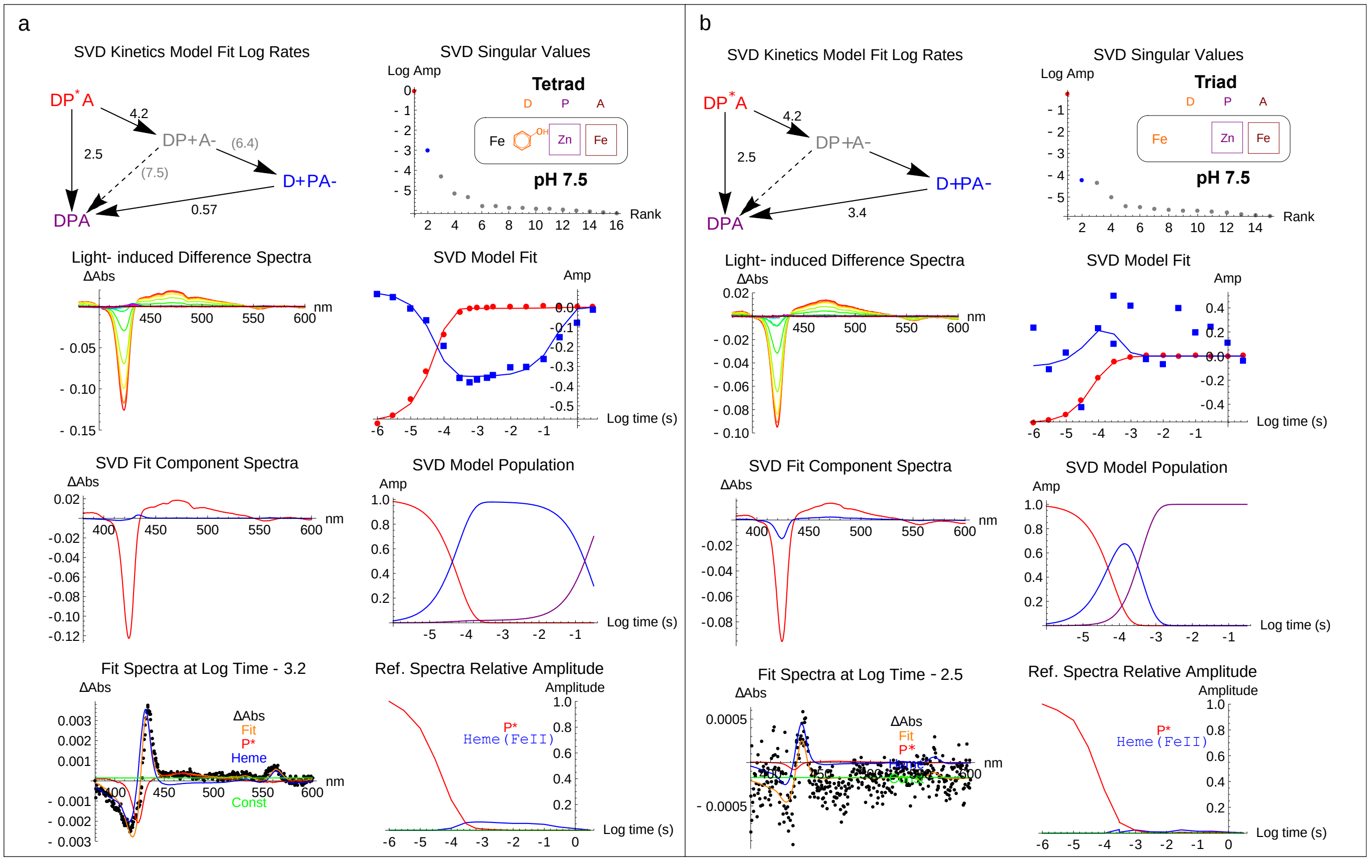

Supplementary Figure 10. Transient absorption kinetics of Fe-Tyrosine-ZnP-Heme Tetrad and Tyrosine knock-out. a. L71H variant at pH 7.5 with ZnP, heme B, and 10 μM Fe(II)Cl_2. SVD indicates a second kinetic species well above the background noise. A 3-state kinetic model in which k A^- to P^+ is much faster than k P to A gives log k P to A of 4.2 s^{-1} and a D^+PA^- spectrum with a clear heme redox signal (blue), providing direct evidence of heme reduction. Decay of the D^+PA^- charge separated state to the ground state has a log rate of 0.6 s^{-1}, significantly slower than the metal-free Supplementary Fig. 8b. The yield of heme reduction relative to the initial P* triplet state (red spectrum) is 6.4%. Using this yield, a 4-state model gives log rates in parentheses. **b.** Y168L mutant was prepared with ZnP, heme B, and 10 μM Fe(II)Cl_2 at pH 7.5. Knocking out the tyrosine donor decouples electron transfer from Fe(II). SVD indicates a second kinetic species close to the background noise. A 3-state kinetic model in which k A^- to P^+ >> k P to A, giving log k P to A of 4.2 s^{-1}. The D^+PA^- spectrum does not have a clear heme redox signal and decay of this state back to the ground state DPA is uncertain, but has a log rate significantly faster than Tyr containing maquette in panel (a), indicating that Tyr facilitates electron transfer from Fe(II).

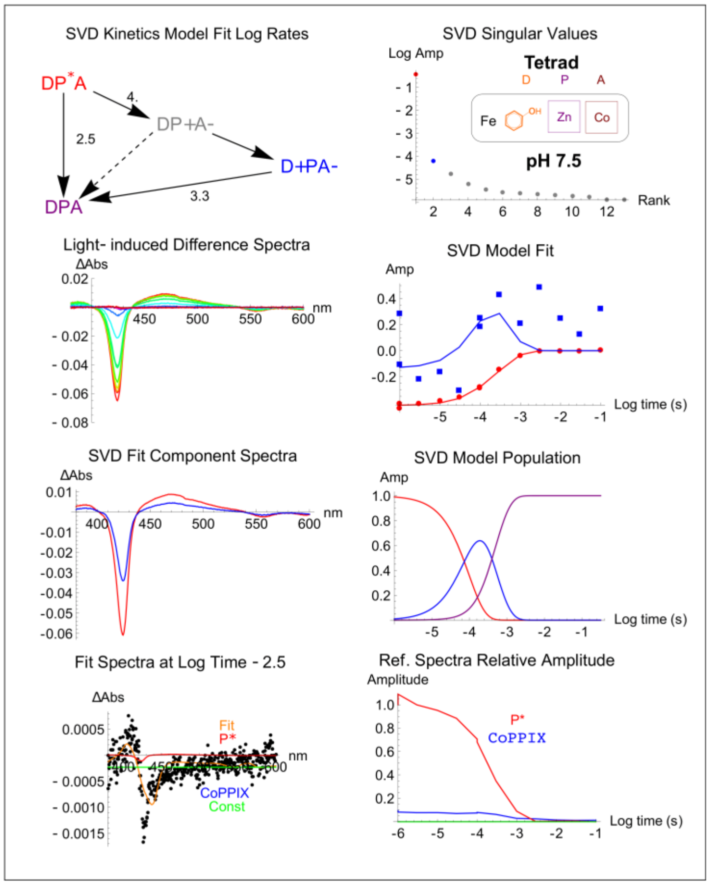

Supplementary Figure 11. Transient absorption kinetics of Fe-Tyrosine-ZnP-CoPPIX Tetrad. At pH 7.5 with 10 μM Fe(II)Cl_2, using the monad ^{3}ZnP* decay rate of 2.5 s^{-1} with a 3-state system gives log k P to A of 4.0 s^{-1}. At long delay times an absorbance blue-shift is evident, as expected for Co(III)PPIX photoreduction to Co(II)PPIX, but the signal is small. An accurate yield estimate is not possible, but spectral fits indicate a yield of a few percent. Final charge recombination of D^+PA^- to the ground state is not well determined. CoPPIX significantly increases the rate of P* quenching compared to the monad (Supplementary Fig. 4a).

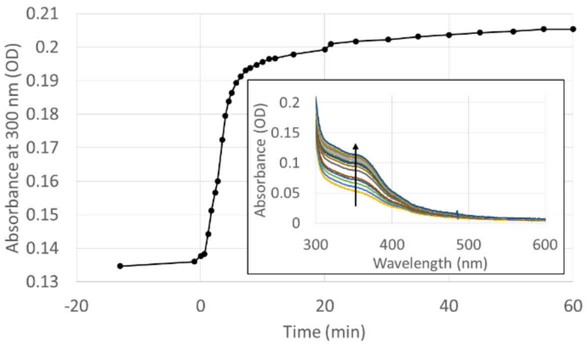

Supplementary Figure 12. Ferroxidase activity in RC maquette. Ferroxidase activity is observed in the RC maquette, demonstrating that the iron center is redox active. An anaerobic solution of 31 µM apo-state RC maquette was prepared with 60 µM Fe(II)Cl_2 in 150 mM KCl and 50 mM MOPS buffer at pH 7.3 in a capped quartz cuvette. At time t=0, ambient oxygen was introduced by removing the cap and stirring. Similar to other diiron proteins, oxygen exposure resulted in the appearance of a ligand-to-metal charge transfer band near 300 nm. The extinction coefficient (on a per iron basis) in the diferric state of the RC maquette is 3400 $M^{-1}cm^{-1}$ at 300 nm, similar to the values of 3240 $M^{-1}cm^{-1}$ and 3380 $M^{-1}cm^{-1}$ for DF2 and bacterioferritin, respectively.[5,6]

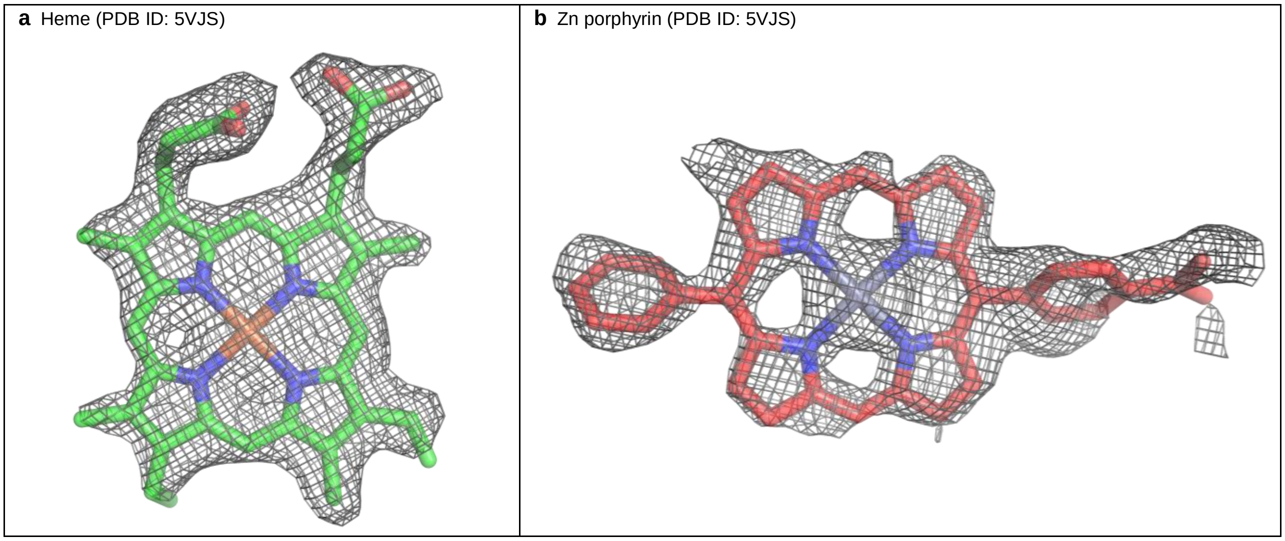

Supplementary Figure 13. Polder omit maps of the heme B and ZnP sites. A polder map is an omit map that excludes bulk solvent close to the omitted region to create an electron density map that removes model bias but has higher quality than a simple omit map. Polder maps were calculated in Phenix, as described in Methods. Maps are presented at 3.0 σ contouring levels (grey mesh). **a.** Polder map of heme B electron acceptor in crystal structure containing ZnP (PDB ID: 5VJS). **b.** Polder map of the ZnP pigment in structure 5VJS shows improved electron density compared to the simple omit map in Supplementary Figure 2d.

Supplementary references

1 Bertini, I. & Luchinat, C. High spin cobalt(II) as a probe for the investigation of metalloproteins. *Adv Inorg Biochem* **6**, 71-111 (1984).

2 Torres Martin de Rosales, R. *et al.* Spectroscopic and metal-binding properties of DF3: an artificial protein able to accommodate different metal ions. *J Biol Inorg Chem* **15**, 717-728 (2010).

3 Keech, A. M. *et al.* Spectroscopic studies of cobalt(II) binding to *Escherichia coli* bacterioferritin. *J Biol Chem* **272**, 422-429 (1997).

4 Wang, Z. X. & Jiang, R. F. A novel two-site binding equation presented in terms of the total ligand concentration. *FEBS Lett* **392**, 245-249 (1996).

5 Pasternak, A., Kaplan, J., Lear, J. D. & Degrado, W. F. Proton and metal ion-dependent assembly of a model diiron protein. *Protein Sci* **10**, 958-969 (2001).

6 Yang, X., Le Brun, N. E., Thomson, A. J., Moore, G. R. & Chasteen, N. D. The iron oxidation and hydrolysis chemistry of *Escherichia coli* bacterioferritin. *Biochemistry* **39**, 4915-4923 (2000).
