# Supplementary material for: De novo protein design of photochemical reaction centers
Source: Nat Commun. 2022 Aug 23;13:4937. doi: 10.1038/s41467-022-32710-5 (PMC9399245; doi:10.1038/s41467-022-32710-5)
Supplement: Supplementary file 3 — Reporting Summary [file 41467_2022_32710_MOESM3_ESM.pdf]

nature portfolio

Corresponding author(s): Nathan M. Ennist

Last updated by author(s): Aug 6, 2022

Reporting Summary

Nature Portfolio wishes to improve the reproducibility of the work that we publish. This form provides structure for consistency and transparency in reporting. For further information on Nature Portfolio policies, see our Editorial Policies and the Editorial Policy Checklist.

Statistics

For all statistical analyses, confirm that the following items are present in the figure legend, table legend, main text, or Methods section.

n/a	Confirmed	
☐	☒	The exact sample size (*n*) for each experimental group/condition, given as a discrete number and unit of measurement
☐	☒	A statement on whether measurements were taken from distinct samples or whether the same sample was measured repeatedly
☒	☐	The statistical test(s) used AND whether they are one- or two-sided<br>*Only common tests should be described solely by name; describe more complex techniques in the Methods section.*
☒	☐	A description of all covariates tested
☐	☒	A description of any assumptions or corrections, such as tests of normality and adjustment for multiple comparisons
☒	☐	A full description of the statistical parameters including central tendency (e.g. means) or other basic estimates (e.g. regression coefficient) AND variation (e.g. standard deviation) or associated estimates of uncertainty (e.g. confidence intervals)
☒	☐	For null hypothesis testing, the test statistic (e.g. *F*, *t*, *r*) with confidence intervals, effect sizes, degrees of freedom and *P* value noted<br>*Give P values as exact values whenever suitable.*
☒	☐	For Bayesian analysis, information on the choice of priors and Markov chain Monte Carlo settings
☒	☐	For hierarchical and complex designs, identification of the appropriate level for tests and full reporting of outcomes
☒	☐	Estimates of effect sizes (e.g. Cohen's *d*, Pearson's *r*), indicating how they were calculated

Our web collection on statistics for biologists contains articles on many of the points above.

Software and code

Policy information about availability of computer code

Data collection	WinSpec/32 (for use with Princeton Instruments PI-MAX3 ICCD camera)
Data analysis	XDS, version October 15, 2015; Phenix version 1.10pre-2124; CCP4i 6.5.019; Coot 0.8.1; PyMOL Version 2.5.0a0 Open-Source; Wolfram Mathematica, version 11.1.1; Phaser; REFMAC5 Version 5.8.0131; PDB_REDO; SOLVE; RESOLVE; SCALA

For manuscripts utilizing custom algorithms or software that are central to the research but not yet described in published literature, software must be made available to editors and reviewers. We strongly encourage code deposition in a community repository (e.g. GitHub). See the Nature Portfolio guidelines for submitting code & software for further information.

Data

Policy information about availability of data

All manuscripts must include a data availability statement. This statement should provide the following information, where applicable:

- Accession codes, unique identifiers, or web links for publicly available datasets
- A description of any restrictions on data availability
- For clinical datasets or third party data, please ensure that the statement adheres to our policy

X-ray crystallographic coordinates and data files were deposited at the Protein Data Bank (PDB) with accession codes 5VJS [http://doi.org/10.2210/pdb5vjs/pdb] (RC maquette with heme B, ZnP, and Zn2+), 5VJT [http://doi.org/10.2210/pdb5vjt/pdb] (RC maquette with heme B and Zn2+), and 5VJU [http://doi.org/10.2210/pdb5vju/pdb] (RC maquette L71H mutant with heme B and Cd2+). Source data for Figure 3 are provided as a Source Data file with the paper.

Human research participants

Policy information about studies involving human research participants and Sex and Gender in Research.

Reporting on sex and gender	N/A
Population characteristics	N/A
Recruitment	N/A
Ethics oversight	N/A

Note that full information on the approval of the study protocol must also be provided in the manuscript.

Field-specific reporting

Please select the one below that is the best fit for your research. If you are not sure, read the appropriate sections before making your selection.

- [x] Life sciences
- [ ] Behavioural & social sciences
- [ ] Ecological, evolutionary & environmental sciences

For a reference copy of the document with all sections, see nature.com/documents/nr-reporting-summary-flat.pdf

Life sciences study design

All studies must disclose on these points even when the disclosure is negative.

Sample size	Each transient absorption difference spectrum at each delay time used for analysis was the average of at least 50 spectra. This sample size was sufficient to ensure reproducibility. Delay times were selected to adequately capture the initial magnitude of the Soret bleach in the triplet state and the transition to the final ground state.
Data exclusions	Data were not excluded from analysis.
Replication	All attempts at replicating X-ray crystallographic and transient absorption experiments were successful. The 3 X-ray crystal structures reported show a similar overall conformation of the protein. Reproducibility of transient absorption measurements at the same delay time at the beginning and end of data collection revealed negligible photodegradation of samples during the experiment. As shown in Supplementary Table 2, each electron transfer rate was measured in independently-prepared samples by transient absorption between 9 and 40 times.
Randomization	Randomization was not relevant to this study. Protein samples were prepared with different cofactors, and controls were prepared without various cofactors. Since all samples and controls were subjected to a standardized transient absorption experiment, there was no group allocation to randomize.
Blinding	Blinding was not relevant to this study. In transient absorption experiments, blinding was not possible because intermediate spectra revealed which chemical species were present; all data sets were analyzed with the same method to eliminate bias. In crystallographic experiments, blinding was not feasible; bias in small molecule identification was removed using omit maps.

Reporting for specific materials, systems and methods

We require information from authors about some types of materials, experimental systems and methods used in many studies. Here, indicate whether each material, system or method listed is relevant to your study. If you are not sure if a list item applies to your research, read the appropriate section before selecting a response.

Materials & experimental systems

n/a	Involved in the study	
x		Antibodies
x		Eukaryotic cell lines
x		Palaeontology and archaeology
x		Animals and other organisms
x		Clinical data
x		Dual use research of concern

Methods

n/a	Involved in the study	
x		ChIP-seq
x		Flow cytometry
x		MRI-based neuroimaging
